# Supplementary material for: Elevated Kappa Index in the Absence of Cerebrospinal Fluid IgG Oligoclonal Bands: Contribution of Intrathecal IgM and IgA Synthesis
Source: Biomolecules. 2025 Jan 9;15(1):90. doi: 10.3390/biom15010090 (PMC11764083; doi:10.3390/biom15010090)
Supplement: Supplementary file 1 [file biomolecules-15-00090-s001.zip › biomolecules-3307945-supplementary.pdf]

Supplementary material S1: Description of laboratory methods

Compared with the methodology described by Villar et al. [11], the main modifications are as follows: agarose isoelectric focusing (IEF) gel is prepared with 1.5 ml of Pharmalyte (pH 5-8) and 2.0 ml of Pharmalyte (pH 3-10); 10 µl paired samples are applied using sodium dodecyl sulphate (SDS) applicator strips; the polyvinylidene fluoride (PVDF) membrane is wetted in ethanol and then washed in two changes of the saline solution; and the membrane is incubated with rabbit anti-human IgM and then with alkaline phosphatase (AP)-conjugated goat anti-rabbit.

Supplementary material S2

Table S1: Frequency of different diagnoses in the overall population and in the Kappa-OCB-, and Kappa+OCB+ patients

**Final diagnosis**	**Overall population (%)**	**Kappa-/OCB- (%)**	**Kappa+/OCB+ (%)**
MS	46 (39)	1 (4)	18 (69)
Miscellaneous*	17 (14)	7 (29)	1 (4)
Infectious encephalitis**	15 (13)	0 (0)	1 (4)
Autoimmune encephalitis***	8 (7)	5 (21)	2 (8)
Epilepsy/status epilepticus	5 (4)	3 (13)	2 (8)
Isolated acute transverse myelitis (non-infectious)	5 (4)	0 (0)	0 (0)
Cerebral lymphoma	3 (3)	1 (4)	0 (0)
Stroke	3 (3)	2 (8)	0 (0)
CIDP + GBS	3 (3)	0 (0)	0 (0)
Unspecified dementia	2 (2)	2 (8)	0 (0)
CVT	2 (2)	1 (4)	0 (0)
CNS vasculitis	2 (2)	0 (0)	2 (8)
Neurosarcoidosis	2 (2)	0 (0)	0 (0)
ALS	2 (2)	2 (8)	0 (0)
MOGAD	2 (2)	0 (0)	0 (0)
Meningeal carcinomatosis	2 (2)	0 (0)	0 (0)

MOGAD: myelin oligodendrocyte glycoprotein antibody-associated disorder; ADEM: acute disseminated encephalomyelitis; ALS: amyotrophic lateral sclerosis; CVT: cerebral venous thrombosis; CIDP: chronic inflammatory demyelinating polyneuropathy; GBS: Guillan–Barré syndrome.

*One case of each of Neuro-Behçet, infectious myeloradiculitis, migraine with aura, Degos' disease, Bickerstaff encephalitis, mitochondrial disease, viral

cerebellitis, Borrelia polyradicultis, acute disseminated encephalomyelitis (ADEM), progressive supranuclear palsy, tension-type headache, intracranial artery stenosis, FAS-associated death domain protein (FADD) mutation leukoencephalopathy, autoimmune chorea, neurosyphilis, neurinoma, and vascular parkinsonism.

**West Nile virus (n=5), unidentified (n=3), one each of SARS-Cov-2, Epstein–Barr virus, enterovirus, herpes simplex 1, HIV, Toscana virus, and Mycoplasma pneumoniae.

***Limbic encephalitis (n=2), anti-GAD encephalitis (n=2), seronegative AE (n=2), and one case each of anti-MA2 encephalitis and anti-NMDAR encephalitis.

Patients were classifed into different diagnostic categories as follows:

INFL: autoimmune encephalitis (n=8), Bickerstaff encephalitis (n=1), ADEM (n=1), MOGAD (n=2), non-infectious myelitis (n=5), Neuro-Behçet (n=1), CNS vasculitis (n=2), neurosarcoidosis (n=2), autoimmune chorea (n=1), CIDP (n=2), and GBS (n=1).

INFECT: infectious encephalitis (n=15), neurosyphilis (n=1), infectious myeloradiculitis (n=1), Borrelia polyradiculitis (n=1), and viral cerebellitis (n=1).

Other: the remaining patients.

Supplementary material S3

Table S2: Results of significant post hoc analyses relative to Table 2.

**Parameter**	**Kappa+OCB- versus Kappa-OCB-**	**Kappa+OCB- versus Kappa+OCB+**	**Kappa-OCB- versus Kappa+OCB+**
Age	<0.010	NS	<0.001
Kappa index	<0.001	<0.001	<0.001
IgG Index	NS	<0.001	<0.001
% of patients with elevated IgG index	0.007	0.002	<0.001
IgA index	<0.010	NS	NS
% of patients with positive IgG/IgM/IgA index or IgM OCBs	NS	NS	0.011

NS: not significant.

Supplementary material S4

Table S3: Results of significant post hoc analyses relative to Table 4.

Parameter	MS versus INFL	MS versus INFECT	MS versus Other	INFL versus INFECT	INFL versus Other	INFECT versus Other
Age	0.009	0.019	<0.001	NS	NS	NS
Sex (male)	NS	0.009	<0.001	NS	NS	NS
Kappa index	<0.001	NS	<0.001	0.023	0.021	<0.001
% of patients with kappa index ≥5.0	0.003	NS	<0.001	0.031	0.031	<0.001
% of patients with elevated IgG index	NS	NS	0.016	NS	NS	0.018
IgM index	NS	<0.001	NS	<0.001	NS	<0.001
% of patients with elevated IgM index	NS	<0.001	0.047	0.001	NS	0.003
IgA index	NS	0.001	NS	0.004	NS	0.004
% of patients with elevated IgA index	NS	<0.001	NS	<0.001	NS	0.003
IgM OCB-positive patients	NS	<0.001	0.012	0.012	NS	NS
% of patients with positive IgM/IgA index or IgM OCBs	NS	<0.001	0.016	0.003	NS	0.007
% of patients with positive IgG/IgM/IgA	NS	0.001	NS	0.007	NS	0.007

index or IgM OCBs						
CSF cell count	0.010	<0.001	NS	0.021	NS	<0.001
CSF proteins	<0.001	<0.001	<0.001	0.033	NS	NS
QAlb x 100	0.002	<0.001	<0.001	NS	NS	NS

NS: not significant.
